# Organisation of the motor cortex differs between people with and without knee osteoarthritis

**DOI:** 10.1186/s13075-015-0676-4

**Published:** 2015-06-18

**Authors:** Camille J. Shanahan, Paul W. Hodges, Tim V. Wrigley, Kim L. Bennell, Michael J. Farrell

**Affiliations:** Department of Physiotherapy, The University of Melbourne, Melbourne, Australia; The Florey Institute of Neuroscience and Mental Health, Kenneth Myer Building, 30 Royal Parade, Parkville, VIC 3052 Australia; Centre of Clinical Research Excellence in Spinal Pain, Injury and Health, The University of Queensland, Brisbane, Australia; Department of Medical Imaging and Radiation Sciences, Monash University, Melbourne, Australia

## Abstract

**Introduction:**

The aim of this study was to investigate possible differences in the organisation of the motor cortex in people with knee osteoarthritis (OA) and whether there is an association between cortical organisation and accuracy of a motor task.

**Methods:**

fMRI data were collected while 11 participants with moderate/severe right knee OA (6 male, 69 ± 6 (mean ± SD) years) and seven asymptomatic controls (5 male, 64 ± 6 years) performed three visually guided, variable force, force matching motor tasks involving isolated isometric muscle contractions of: 1) quadriceps (knee), 2) tibialis anterior (ankle) and, 3) finger/thumb flexor (hand) muscles. fMRI data were used to map the loci of peak activation in the motor cortex during the three tasks and to assess whether there were differences in the organisation of the motor cortex between the groups for the three motor tasks. Root mean square of the difference between target and generated forces during muscle contraction quantified task accuracy.

**Results:**

A 4.1 mm anterior shift in the representation of the knee (*p* = 0.03) and swap of the relative position of the knee and ankle representations in the motor cortex (*p* = 0.003) were found in people with knee OA. Poorer performance of the knee task was associated with more anterior placement of motor cortex loci in people with (*p* = 0.05) and without (*p* = 0.02) knee OA.

**Conclusions:**

Differences in the organisation of the motor cortex in knee OA was demonstrated in relation to performance of knee and ankle motor tasks and was related to quality of performance of the knee motor task. These results highlight the possible mechanistic link between cortical changes and modified motor behavior in people with knee OA.

## Introduction

Along with pain and changes to knee joint tissues (cartilage, bone, ligaments, muscles and joint capsule), changes in sensory and motor function of the knee are common in people with knee osteoarthritis (OA) [1], yet the underlying mechanisms are not completely understood. It is possible that these changes may be mediated by alteration to the motor regions of the cortex of the brain. Most people with knee OA symptoms experience some degree of impaired motor function [2, 3]. Changes in motor control in knee OA include: alterations to gait and muscle activation patterns [4, 5], quadriceps muscle weakness [6] and impaired proprioception [7]. Altered organisation of the motor and sensory regions of the cerebral cortex accompanies modified motor control in a range of other musculoskeletal conditions such as recurrent low back pain [8, 9], lateral epicondylalgia [10] and focal hand dystonia [11]. Differing organisation of the brain motor region associated with knee OA is plausible for a number of reasons: 1) the fundamental role of cortical motor regions for the control of movement (including control of basic functions such as gait [12]), 2) the relationship between motor cortex changes and modified behaviour [8], 3) the spectrum of changes to motor control in knee OA [4–7] and, 4) the presence of motor cortex changes in other musculoskeletal conditions [8–11]. To the best of our knowledge there have been no previous studies examining the organisation of the motor cortex in people with knee OA.

The adult brain maintains the ability to reorganise in response to activity, injury, stimulation or learning [13]. Reorganisation of the brain involves neural plasticity, which refers to capacity of the nervous system to change morphologically and/or functionally in association with changes in experience [13, 14], although the cause and effect relationship between brain changes and changes in experience is not always clear. In people with knee OA, the changes to motor control constitute significant changes to experience, which could involve brain reorganisation. Reorganisation within the somatosensory and/or motor cortex is often characterised by changes to the somatotopic representation in the sensory and/or motor homunculi [13]. Such reorganisation has been characterised by contraction or expansion of the representation of the affected body part, accompanied by the contraction, expansion or overlap of adjacent representations of other body parts [15, 16]. Expansion of the face representation into the contracted hand representation following hand amputation is an example of this [15, 16]. Somatotopic reorganisation has also been demonstrated in back and upper limb pathologies as overlap between the normally discrete areas of motor cortex that control upper limb [10] or back muscles [17]. In general, there is minimal or no reorganisation of cortical representations at sites that control separate functions and are spatially separated from the primary affected area [18–20]. Although the knee is the primary site of changes to motor control in knee OA, many features of the adapted motor control involve complex functions with interaction between multiple body segments. Reorganisation of motor cortex representations of adjacent lower limb segments, but not the upper limb, is plausible.

Functional magnetic resonance imaging (fMRI) of brain activity has been used extensively to investigate organisation and reorganisation of the motor cortex and other areas of the brain related to a range of neurological and orthopaedic conditions [14]. fMRI evaluates neural activity from change in blood flow related to the energy use of neurons using the blood oxygenation level dependent (BOLD) signal [21]. Differences in BOLD signal have been observed with neuroplastic changes related to experience [13, 14], such as following limb amputation [15, 16], and provides an ideal method to study potential changes in brain activity in knee OA.

The relevance of differences in brain organisation for motor function depends on identification of a relationship with behaviour. Such a relationship has been identified in some [17], but not all [10] conditions. Our objective was to investigate the potential for modified motor cortex organisation using fMRI, during a motor task known to be modified in knee OA. It was essential that the task could be performed using an MRI scanner and in an identical manner at several different body segments. An ideal solution was performance of a force-matching task, which exhibits reduced accuracy of matching when performed with knee muscles in knee OA [22]. We hypothesised that people with knee OA would differ from controls with respect to distribution/location of active regions of the motor cortex during force-matching at the knee and during force-matching at the ankle, but not during force-matching at the hand. We further hypothesized that the degree of difference in organisation of the motor cortex would be related to quality of performance of the motor task.

## Methods

### Participants

Eleven individuals with moderate/severe right knee OA (six male, five female) and seven healthy asymptomatic controls (five male, two female) participated in the study. Age, height, mass and body mass index did not differ between groups (see Table 1). All participants were right-foot dominant or had no foot preference based on the revised Waterloo footedness questionnaire [23].

**Table 1:** Participant characteristics

Participant characteristic	Osteoarthritis group	Control group	Test for group difference (independent *t* test)
	Mean ± SD	Mean ± SD	
Age (years)	68.9 ± 6.4	64.0 ± 6.7	*p* = 0.14
Height (m)	1.7 ± 0.9	1.7 ± 0.9	*p* = 0.50
Mass (kg)	76.5 ± 12.80	72.1 ± 13.4	*p* = 0.56
Body mass index (mass/height^2^)	27.4 ± 3.7	24.8 ± 2.7	*p* = 0.13

Participants with knee OA had tibiofemoral joint OA in the right knee fulfilling the American College of Rheumatology (ACR) classification criteria [24], had an average intensity of knee pain ≥3 on an 11-point numeric rating scale (anchored with no pain at 0 and worst pain imaginable at 10) on most days of the month prior to enrolment (group mean 4.3 ± 0.8). The Western Ontario and McMaster Universities Arthritis Index (WOMAC) [25] was administered to OA participants (scored 0–96; higher scores on the WOMAC indicate worse pain, stiffness, and functional limitations) (group mean 30 ± 13). OA participants were included if they had a Kellgren and Lawrence (KL) grade of 3 or 4 on weight-bearing x-ray (n = 9 with KL grade 4) [26]. KL grading/screening was performed by one of two researchers trained in the KL grading system, using radiographs taken within 12 months of the participant’s enrolment in the study. Participants were included in the control group if they had no knee pain or knee injury in the past 5 years. Volunteers were excluded from either group if they had signs or symptoms of other conditions, including co-existing conditions that could hinder participation or performance of the experimental task (e.g., neurological conditions, lower limb surgery, upper limb pain or surgery, or systemic arthritis). The University of Melbourne Human Research Ethics Committee approved the study and all participants provided written informed consent.

### Procedure

**Fig. 1:**
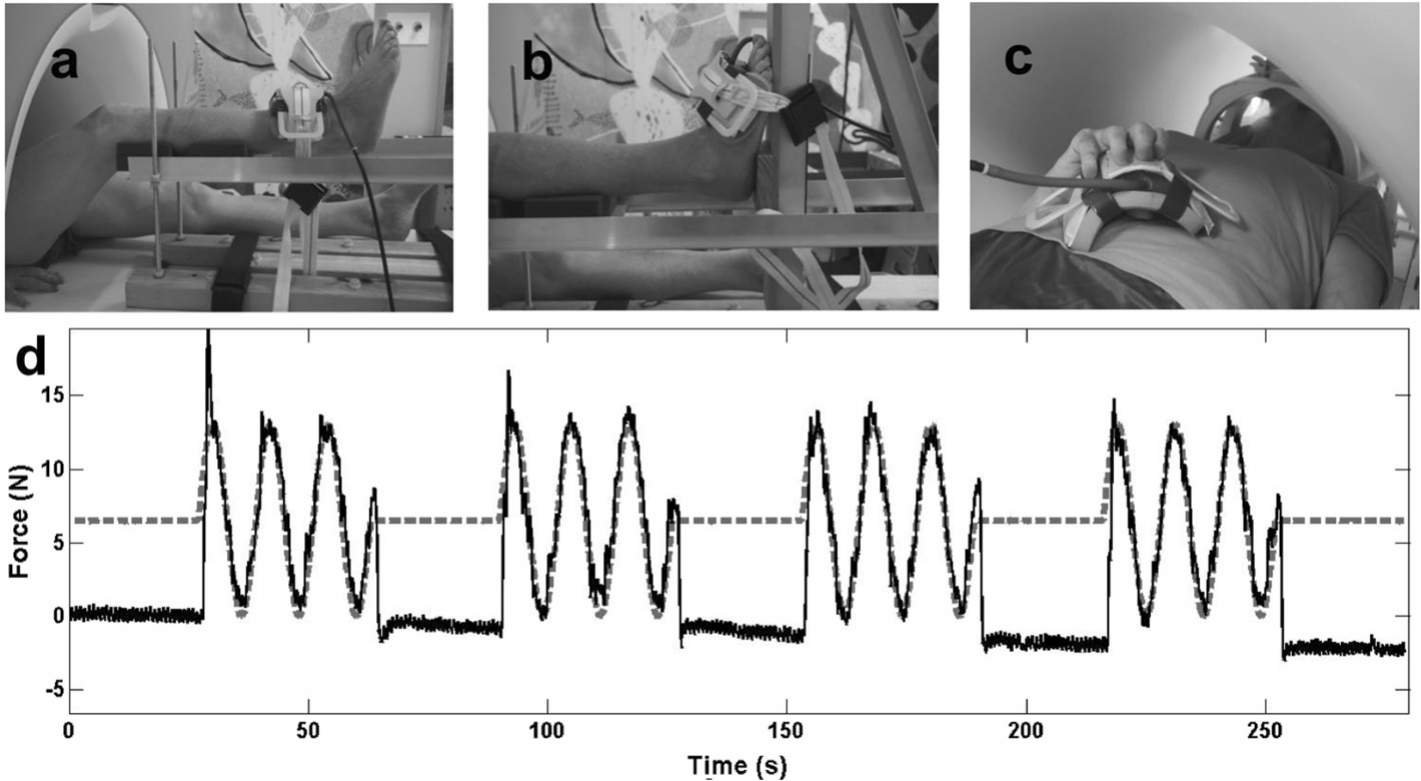
Experimental setup. A control participant with the force recording apparatus used for **a** knee (quadriceps), **b** ankle (tibialis anterior), and **c** hand force-matching tasks. **d** Force-matching output: target force (*grey dashed line*) and match force (*black line*) output for a control participant’s knee task (representative of output across all participants and tasks)

fMRI data were collected while participants performed three visually guided force-matching tasks that involved submaximal isometric contractions of the quadriceps (knee task), tibialis anterior (ankle task) or hand (hand task) muscles in the order randomised. Participants lay supine in the MRI scanner with their leg supported on custom-made MRI-compatible apparatus that isolated contractions of the quadriceps or tibialis anterior. The leg was also positioned in the apparatus during performance of the hand muscle task. The experimental setup included an adjustable rig [27] (similar to that described previously [28]) and force measurement apparatus (see Fig. 1). The MRI-safe rig made of wood, aluminium and plastic stabilised the lower limb as the participant exerted isometric knee extension or ankle dorsiflexion. The force measurement apparatus consisted of a strap, sphygmomanometer cuff and pressure transducer unit (Vernier Scientific GPS-BTA, Beaverton, OR, USA). The strap was made of aluminium, webbing material and plastic, and was used to secure the participant’s lower limb to the rig. A small sphygmomanometer cuff was placed between the strap and the limb to measure force production. The pressure cuff was linked via air-tight plastic tubing to the pressure transducer unit housed outside the MRI room. The strap was placed over the ankle (for quadriceps) or dorsum of the foot (for tibialis anterior) or between the thumb and fingers (for hand muscles) (see Fig. 1). Prior to testing, calibration with certified weights was used to established equations to derive force from the pressure recordings. This was used to provide real-time display of force from the recorded pressure data using custom Matlab software (Mathworks, USA) and a DI-158 USB analogue-to-digital converter (DATAQ, Akron, OH, USA).

A block design was used that involved four contraction blocks interleaved with five rest blocks. The duration of rest blocks was 27 s, during which time participants exerted no force. Contraction blocks lasted 36 s and involved isometric contractions to follow a sinusoidal force target (generated by a Hewlett-Packard 33120A digital frequency generator and projected via a rear-projection screen and head coil-mounted mirror viewing system) that guided modulation of contraction intensity from 0–5 % of maximal voluntary contraction (MVC) in a sinusoidal pattern with a cycle duration of 12 s for three cycles. A force target of 5 % MVC was used to tailor contraction intensity to each participant’s strength and was found in preliminary testing to activate the motor cortex without movement of the head in a manner correlated with the motor task (10 % MVC-produced correlated head movement). Target and feedback of the participant’s force were displayed in an overlayed manner and participants were instructed to match the target force as accurately as possible (Fig. 1d shows force-matching output). A trigger signal from the MRI scanner started each sequence of the protocol and enabled synchronisation of the MRI and force data. Participants were familiarised with the force-matching protocol on two occasions: first, in a session in the movement laboratory in the months prior to the MRI testing session, and second, in an MRI mock scanner immediately preceding the MRI session. The force-matching task was specifically designed and tested to minimise the likelihood of provocation of knee pain. During testing of the protocol prior to the MRI session, pain was rated following the knee task using the 11-point numeric rating scale. All participants with OA rated their knee pain following performance of the knee task at an intensity lower than that during the month prior to study enrolment. No control participants experienced pain following the knee task.

Force generated during MVC efforts for each site was established immediately prior to MRI testing using the same experimental setup in a mock MRI scanner. Two submaximal warm-up efforts were followed by three MVC efforts lasting for 3 s, each interspersed with 30 s rest periods. Participants were verbally encouraged to reach maximum. The highest peak force of the three trials was used to calculate force targets.

Accuracy/quality of performance of the force-matching tasks was measured as the root mean square (RMS) of the difference between target and generated forces (RMS error) during each of the four contraction blocks. Mean root mean squared (RMS) error (% MVC) was calculated as the average over the four contraction blocks.

### Brain imaging

#### Acquisition

fMRI scans were acquired using a Siemens Trio 3T (Munich, Germany) MRI machine with a 32-channel head coil. Head movement was restricted using tight-fitting headphones and foam padding placed between the participant’s head and the MRI coil. For each of the three force-matching tasks echo-planar images (EPI), using BOLD contrast, were acquired in the transaxial plane (40 slices; 3 mm thickness; 1.95 × 1.95 mm^2^ in-plane resolution; echo time (TE) 35 ms; repetition time (TR) 3,000 ms; flip angle 90 °) during scans of 4.8 minutes duration that incorporated 96 sequential images. Structural T1-weighted images were acquired in the sagittal plane (192 slices; 0.90 mm thickness; 0.84 × 0.84 mm in-plane resolution; TE 2.95 ms; TR 1,900 ms; flip angle 90 °).

#### Processing

All fMRI processing was performed using FSL 4.1 software [29] and well-established protocols [30] to analyse the BOLD signals related to the motor tasks. Pre-processing and analysis of functional images was performed with FEAT, v5.98 [29]. Pre-processing included registration, motion correction and spatial smoothing (3 mm full width half maximum (FWHM)). Nonlinear registrations of participants’ structural images to the Montreal Neurologic Institute (MNI) 1- mm template were performed. Regressors for contraction and rest blocks using the target force data for each participant for contraction blocks and zero force for rest blocks were included in a general linear model (GLM) that also included motion correction parameters as confound regressors. The fit of the BOLD signal variance to the regressors was represented as parameter estimates. Contrasts of parameter estimates (COPEs) were subsequently performed to contrast between model fit and the null (no fit). These COPEs were then expressed as *z* statistics. The analysis was performed for every voxel (i.e., voxel-wise analysis) and generated statistical parametric maps of signals that correlated with the target force.

FMRI Image Laboratory Linear Registration Tool (FLIRT) [29] registration procedures were used to register the functional image to the structural image, and the structural image to the standard image. These procedures transformed the *z* statistic images from individual to standard space, producing *z* statistic images for each participant for each task (knee, ankle, hand) in standard space, allowing analysis of individual participant’s activations in standard space.

**Fig. 2:**
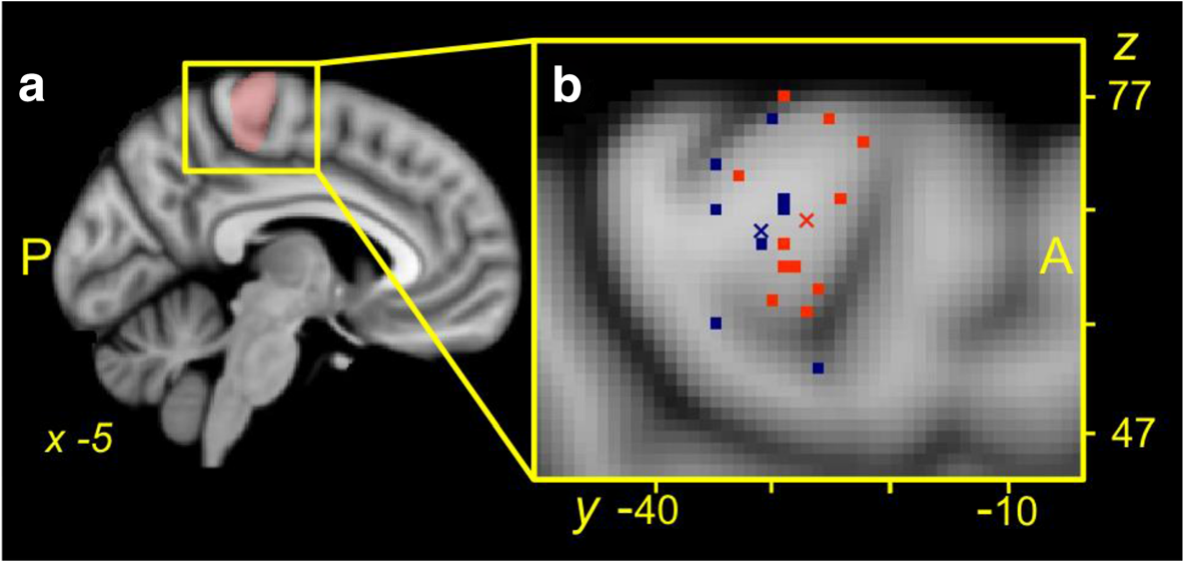
Location of peak motor cortex activation. **a** Location of the anatomical search territory for knee and ankle task peak activations: *pink shaded* voxels. **b** Individual participant (*squares*) and group mean (*x*) loci of peak activation for the knee task collapsed across the *X* (medial/lateral) dimension for participants in the knee osteoarthritis (OA) (*orange*) and control (*blue*) groups. Data are presented on a Montreal Neurologic Institute (MNI) 1-mm template standard brain. Note that the group mean loci of peak activation for the OA group is anterior to that for the control group (*p* = 0.02), and the wide distribution of loci of peak activation for the knee task between individual participants across the search territory of the motor cortex (13 mm in the *Y* plane and 24 mm in the *Z* plane)

Using the *z* statistic image for each participant for each task (knee, ankle and hand), the site of peak activation in the primary motor cortex (M1) for each participant for each task was determined using the following method: 1) the site of peak activation within the contralateral motor cortex was defined as the voxel with the highest *z* value within a cluster of ≥20 contiguous voxels with *z* >2.3 (*p* <0.01); 2) for the knee/ankle area the search area of the motor cortex was defined based on histological demarcation of the Brodmann area 4 [31], as the territory bounded by the medial frontal gyrus (anteriorly), the post central gyrus (posteriorly), the cingulate gyrus (inferiorly), the superior brain margin (superiorly) and from the mesial wall of the left hemisphere to the lateral border of the post central sulcus (mediolaterally). For the hand area the search area was defined as the territory bounded by the medial frontal gyrus (anteriorly), the post central gyrus (posteriorly), and corresponding with the inferior genu as detailed previously [32]; 3) masks of these territories were generated (Fig. 2, knee/ankle mask); 4) the MNI (*X, Y, Z*) coordinates of the voxel with the highest *z* value (peak voxel) within the motor cortex mask was located using custom programming with the *fslstats* function [29]. 5) The location of the peak voxel was verified manually to ensure its location was within the expected area of the motor cortex on the MNI standard 1-mm brain and within a contiguous cluster of ≥20 voxels; 6) as there is individual variation in cortical anatomy, the location of the peak voxel was verified manually to ensure it was within the motor cortex of the participant’s brain images (nonlinear registration of high resolution to MNI 1-mm standard brain) and within a contiguous cluster of ≥20 voxels.

### Statistical analysis of force data and fMRI activation loci

Significance was set at *p ≤*0.05 with level of significance corrected for all multiple comparisons. Data are presented as mean (standard deviation) throughout the text and figures unless otherwise stated.

#### Location of peak motor cortex activation

To assess whether the locations of peak activation in the motor cortex differed between groups (knee OA vs control) for any of the three motor tasks (knee vs ankle vs hand) in any of the three planes, separate 2 × 3 mixed analysis of variance (ANOVA) (group x task) was performed for each image plane (*X, Y* and *Z*).

Post hoc analysis was conducted using four *t* tests with Holm-Sidak correction for multiple comparisons to assess: 1) whether the loci of peak activation differed between the groups for any of the three motor tasks (knee, ankle or hand) and 2) whether the distance between the loci of peak activation for the knee and ankle differed between the groups. The distance (in the anterior-posterior dimension) between the loci of peak activation for the knee and ankle was defined as ankle peak coordinate minus knee peak coordinate for each participant. The corrected *p* value (Holm-Sidak-corrected) for four post hoc tests for the first ranked comparison was *p ≤*0.01 and *p ≤*0.02 for the second ranked comparison.

#### Relationship between loci of peak motor cortex activation, force-matching accuracy and pain

As RMS error data were skewed these data were log transformed. In tasks where locations of peak activation differed between groups, linear relationships between the locations of peak activation and the accuracy of performance of the force-matching tasks (logRMS error) were assessed using Pearson’s correlations, both for data with the groups combined and separately by group. To assess whether clinical knee pain (i.e., average pain on most days of the month prior to enrolment) predicted variance in the location of peak activation in the motor cortex, independently of any variance explained by accuracy of performance of the force-matching task (log RMS error), stepwise multiple regression analysis was used. With location of peak activation in the motor cortex as the dependent variable, pain was entered at the first step and accuracy of performance of the force-matching task was then included in the prediction model. Statistical analyses were performed in SPSS Statistics Version 20 (IBM Company and SPSS Inc., Armonk, NY, USA).

One participant in the OA group was found to have a target force greater than 5 % MVC for the knee force-matching task. All analyses were performed with and without this participant’s data to assess the impact on the group results. As all statistical analyses with or without this participant produced results in the same direction with equal or greater significance, data for this participant have been retained in the analysis*.*

## Results

### Locations of peak motor cortex activation

**Fig. 3:**
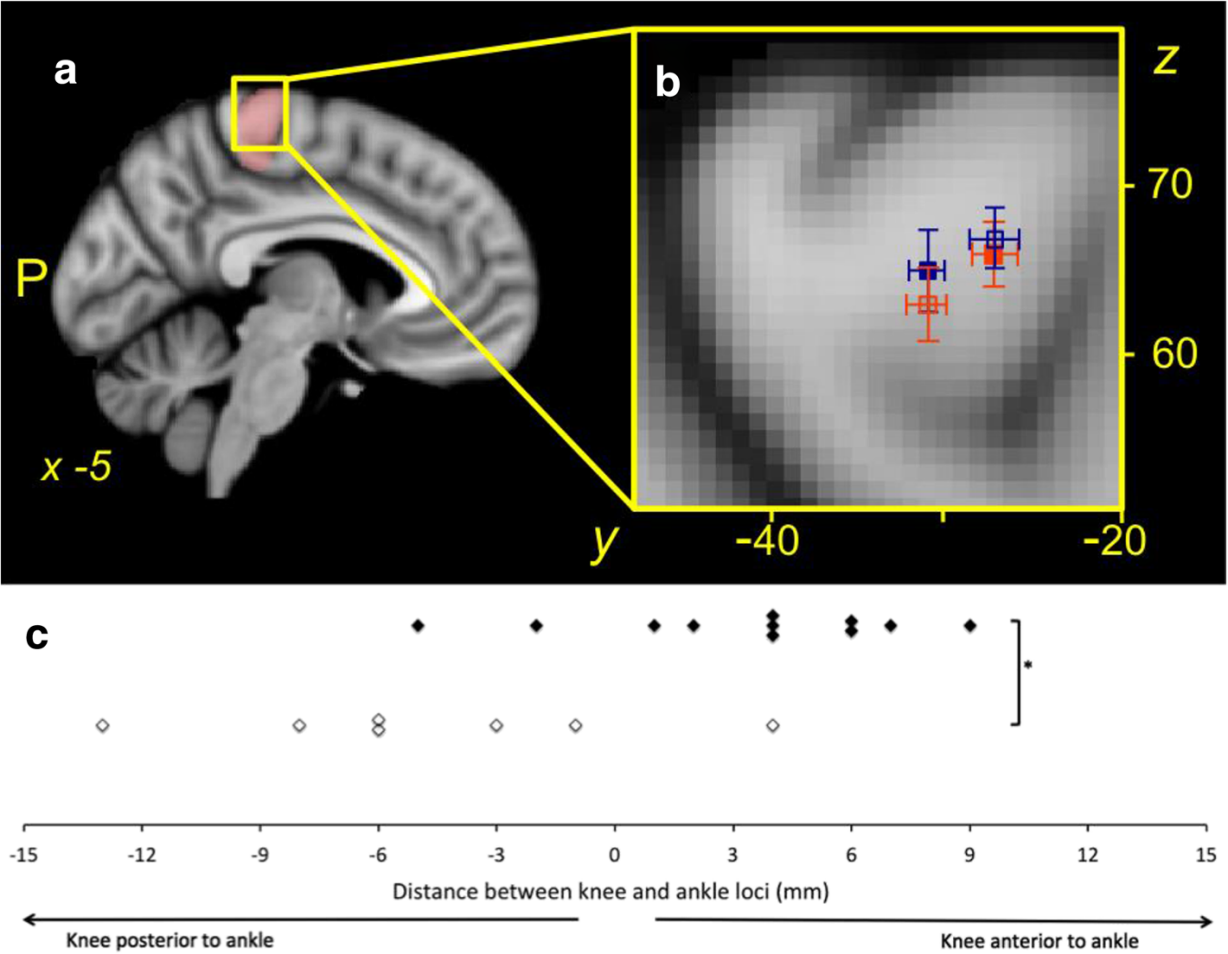
Relative location of the peak motor cortex activation during the knee and ankle tasks. **a** Anatomical search territory for knee and ankle task peak activations: *pink shaded* voxels. **b** Group mean and standard error in the *Y* and *Z* planes are shown for the loci of peak activation for the osteoarthritis (OA) group during the knee (*solid orange square*) and ankle tasks (*open orange* ***s*** *quare*), and for the control group during the knee (*blue solid square*) and ankle (*blue open square*) tasks. **c** Relative location of the sites of peak activation during the knee and ankle tasks for individual participants in the OA (*black*) and control (*white*) groups. Note that the group mean loci of peak activation for the knee task is anterior to that for the ankle task for the OA group, but posterior to that for the ankle task for the control group. This relationship was also observed in the individual data for all but two participants in the OA group, and all but one participant in the control group; *p <0.01 for the between-group difference in the relationship between the knee and ankle loci of peak activation

Knee, ankle and hand force-matching tasks produced significant levels of activation of the motor cortex during the force-matching task in the corresponding somatotopic regions of the contralateral brain hemisphere for all participants. The mean *X, Y, Z* coordinates of the loci of the voxel with the highest level of activation related to the task within the knee/ankle or hand search territory for the corresponding force-matching task for each group are presented in Table 2. Individual and group mean loci for peak motor cortex activation in the search territory during the knee task are shown in Fig 2. Figure 3 shows the mean (standard error) of the loci of peak activation for the knee and ankle tasks.

**Table 2:** *X, Y, Z* coordinates (mean ± SD) of the location of peak activation for the force-matching tasks

		Montreal Neurologic Institute coordinates		
Task	Group	*X*	*Y*	*Z*
Knee	Osteoarthritis	−6.0 ± 3.7	−27.2 ± 3.0*	66.1 ± 6.8
	Control	−5.0 ± 2.8	−31.3 ± 3.4	65.3 ± 6.0
Ankle	Osteoarthritis	−4.7 ± 3.1	−30.5 ± 3.7	62.7 ± 6.1
	Control	−5.5 ± 3.4	−27.5 ± 4.0	67.0 ± 4.7
Hand	Osteoarthritis	−37.6 ± 7.9	−12.4 ± 6.4	57.4 ± 6.0
	Control	−31.2 ± 6.1	−15.6 ± 6.4	60.6 ± 7.1

*P = 0.02 for between-group comparison

Investigation of the interaction between group and site in the *Y* (anterior/posterior) plane (*F* (2, 32) = 4.10, *p* = 0.03) revealed a significant difference between groups in the location of peak activation for the knee task (*t* (16) = 2.62, *p* = 0.02), but not for the ankle or hand (both *p* >0.05). For the knee task the group mean loci of peak activation for the OA group was 4.1 mm more anterior (closer to the medial frontal gyrus) than that of the control group for the same task (Fig. 2). There were no significant interactions between group and site in the *X* (medial/lateral; group x site, *p* = 0.15) or *Z* (proximal/distal; *p* = 0.16) planes.

Further investigation of the interaction between group and site in the *Y* plane revealed that the relative location of the site of peak activation for the knee and the ankle differed between groups. The mean location of peak activation during the knee task was posterior (−4.7 ± 5.4 mm, *t* (16) = −3.58, *p* = 0.003) to that during the ankle task for the controls, but anterior (3.3 ± 4.1 mm) to that during the ankle tasks for participants with knee OA.

### Associations between location of peak motor cortex activation, force-matching accuracy and pain

**Fig. 4:**
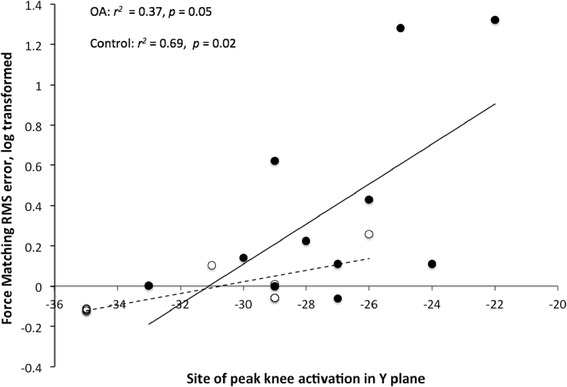
Linear correlation between loci of peak activation and accuracy of performance for the knee force-matching task. Linear correlation between loci of peak activation during the knee task in the *Y* (anterior/posterior) plane and the accuracy of performance of the knee force-matching task. Data are shown for the osteoarthritis (*OA*) (*black marker* and *solid line*) and control (*white marker* and *dashed line*) groups. Note the significant positive correlation between variables for both groups

Force-matching accuracy for the knee task was correlated with location of peak activation in the motor cortex when the data were analysed with both groups combined and when data for each group were analysed separately. The positive linear correlation between accuracy of performance of the knee force-matching task and the loci of peak activation in the *Y* plane (all participants: *r*^*2*^ = 0.44, n = 18, *p* <0.01; knee OA group: *r*^*2*^ = 0.27, n = 11, *p* = 0.05; control group: *r*^*2*^ = 0.69, n = 7, *p* = 0.02; Fig. 4) indicated greater inaccuracy of force matching was related to more anterior location of the site of peak activation.

With knee pain (average pain on most days of the month prior to enrolment) and accuracy of performance of the knee force-matching task included in the predictive model for loci of peak activation in the *Y* plane for the knee task only accuracy of performance of the knee force-matching task predicted loci of peak activation in the *Y* plane for the knee task. The model was significant (*F* (1, 9) = 5.26, *p* = 0.05) and predicted 37% of the variance in the *Y* plane of activation loci.

## Discussion

These data provide novel evidence that organisation of the motor cortex differs in knee OA. These differences in organisation presented as an anterior shift of the knee representation and a switching of the relative anterior-posterior arrangement of the knee and ankle representations in people with knee OA relative to the data for asymptomatic controls. These differences in organisation were identified during performance of a specific motor task previously found altered in knee OA [22]. The amplitude of anterior shift in the motor cortex representation of the knee in knee OA was related to the quality of performance of the task (more anterior representation was related to poorer performance) and was specific to the knee; organisation of the ankle or hand representations in the motor cortex did not differ when an identical task was performed at these segments.

### Differences in the organisation of the knee representation at the motor cortex

The more anterior location of the site of peak motor cortex activation during the knee tasks for the OA group relative to the site identified for controls represents substantial remodelling of this brain region. This difference of around 4.1 mm is approximately 25% of the total anterior-posterior dimension of the motor representation of the knee/ankle (approximately 16 mm) as defined on a standard MNI brain based on histological demarcation of the Brodmann area 4 [31]. It is also a similar order of magnitude to the remodelling of the motor cortex found in low back pain – approximately 15 mm posterior shift in the representation of the *longissimus erector spinae* muscle in the back representation [17], given the trunk representation is larger than that of the knee and ankle [17, 32]. This distance is substantial in the context of mechanisms that are theorised to underpin cortical remodelling at the neuron/synapse level on a microstructural scale [13].

Consistent with previous investigations of motor representations in the cortex, there was considerable variation in the site of peak activity during the knee task in people with and without knee OA [17, 33, 34]. Individual variation in functional localisation of muscles or body parts at the motor cortex is thought to reflect subtle individual differences in the interleaved arrangement of neurons related to specific action within the larger motor representation [34]. Notably, the results of the statistical analysis indicate that the inherent variation within groups was less than the mean difference in location between groups. This implies that the effect of knee OA was greater than small differences between individuals. It is considered that distributed neuronal organisation over a wide cortical area provides a mechanism that limits the disruption to function as a consequence of localised lesions in the motor cortex (e.g., stroke) [35]. Our data show that the distribution of neuronal function related to knee and ankle motor control is affected by peripheral disease. It has previously been indicated that the shifts in motor representations are unlikely to represent a change in location of the cortical pyramidal neurons that output to the motoneurons of the target muscles of interest, but rather a change in the organisation of the network of cortical neurons that input to the pyramidal neurons [13, 17]. Such change in representation may therefore represent modification of synaptic inputs [13].

Reorganisation of the motor cortex is associated with a range of musculoskeletal conditions, although specific features of rearrangement of representations differ between conditions. The differences in the organisation of the motor cortex found in the current study involved a more anterior site of peak activation during the knee task in the OA group, relative to the asymptomatic controls, and switching of relative location of the knee and ankle representations between the OA and control groups. The switching of location of the knee and ankle representations implies differences in the arrangement of the neuronal assemblies related to knee and ankle function between the groups, and is indicative of plastic change in the motor cortex associated with knee OA. Although other studies have demonstrated reduced separation between adjacent cortical regions in low back pain [17] and lateral epicondylalgia [36], and shift in a specific direction [8], these are the first data to show a switching of location with the representation of an adjacent body region/segment.

### Implications of differing cortical organisation for symptoms and treatment of knee OA

The different cortical organisation demonstrated in the current study highlights the possibility that plastic changes in the motor cortex may be caused by knee OA, as an example of activity-dependent plasticity. Alternatively, it is possible that the more anteriorly placed knee representation is a precursor to knee OA symptoms as a consequence of modified joint loading secondary to modified motor patterns [5], which are a risk factor for knee OA (Hodges et al, 2015 unpublished data). Plasticity can be both associated with enhanced performance (e.g., expanded cortical representation of hand muscles in musicians [37]) or compromised performance (e.g., smudging of adjacent finger sensory representations in focal dystonia (writers cramp) [38]). Maladaptive plasticity has been linked with chronic pain, motor impairment and reduced function in a number of chronic pain and musculoskeletal conditions including phantom limb pain [39, 40], low back pain [8], and focal hand dystonia [11]. Cortical reorganisation can vary greatly among individuals despite similar pathology, and the degree of reorganisation has been linked to intensity of pain/dysfunction in people with phantom limb pain [39, 40], spinal cord injury [20] and duration of symptoms in low back pain [9]. As people with radiographic signs of knee OA can present with or without pain [41], this would provide an opportunity to study the relationship between biomechanical function of the knee, pain and reorganisation of the cortex.

The presence of differing cortical organisation in knee OA suggests this may be a potentially modifiable target for treatment of knee OA. Links between the normalisation of cortical organisation and pain reduction and/or improved physical function have been demonstrated in the treatment of other musculoskeletal conditions [39, 42, 43]. Physical therapy techniques including motor retraining, manual and electrophysical therapies such as non-invasive brain stimulation [36, 44], have been shown to modify cortical changes in conjunction with reduced pain and/or improved physical function in musculoskeletal conditions [45, 46]. Further research is required to determine whether cortical representation can be changed and whether it is associated with positive clinical outcomes in knee OA.

### Relationship between differing cortical organisation and motor performance

The relationship between reorganisation of the motor cortex and modified motor behaviour in musculoskeletal conditions has led to speculation about the functional significance of the cortical changes. First, less well-defined cortical representations of adjacent segments/muscles (e.g., greater overlap (so-called smudging) or reduced separation of adjacent representations) in low back pain [17] lateral epicondylalgia [10] and focal hand dystonia [47] are related to reduced capacity for independent control of the individual segments/muscles. It is tempting to speculate that the blurring of the cortical representations underpins the compromised inter-muscle/inter-segment coordination, given that somatotopically discrete centres are considered to be important for fine individuated movement (based on studies of upper-limb control) [47].

Second, motor cortex reorganisation in low back pain has been suggested to reflect adoption of an altered movement strategy to protect the painful part from further pain/injury [17, 48] as has been suggested in contemporary theories of motor adaptation in pain [49, 50]. It is possible that the reorganisation of the motor cortex in knee OA demonstrated here might represent a new motor strategy to protect the part from further injury/pain. It has been argued that altered movement and muscle activation in knee OA may aim to avoid pain and/or stabilise the knee during function [5]. Further work is required to study a possible relationship between the changes identified here and protective features of motor adaptation.

Our data provide evidence of a relationship between features of motor behaviour and brain organisation; more anterior placement loci of peak activation for the knee task was related to less accurate performance of the knee force-matching task. This relationship was demonstrated across the entire group (and each group separately), which strengthens the argued relationship between cortex organisation and behaviour. The basis for this link between brain activation and function is beyond the scope of the current study.

### Hand and ankle motor representation

As expected, the location of peak activation during the hand task did not differ between the groups. This concurs with previous studies that show no change in organisation of representations of body regions at a distance to the primary site of reorganisation [18–20]. Although the absolute position of the ankle representation was not found to be modified significantly with the present sample size, its location relative to the knee differed between groups; the relative position of ankle and knee swapped in the anteroposterior direction. The significance of this change is unclear, but may relate to altered coordination between adjacent segments, as alluded to earlier. Such changes may not be surprising considering the interaction between the ankle and knee in many lower limb functions, and the proximity of the somatotopic representations [18, 19]. Although the absolute position of peak activation during the ankle task was not different, assessment of raw data in Fig. 2 suggests a tendency towards the opposite shift of the ankle representation in the OA group relative to the controls (i.e., more posterior and inferior). A posterior/inferior shift of ankle would be expected if the ankle representation expanded to occupy the anteriorly shifted knee representation and the ankle representation most likely placed superior to the knee representation in normal homuncular arrangements [28, 51]. The failure of this difference to reach significance may not be surprising considering the less well-documented homuncular configurations of the motor cortical representations of the lower limb segments than those of the upper limb, face and trunk, and evidence of substantial overlap between the ankle and knee motor and sensory representations [28, 51].

### Methodological issues

A number of methodological limitations require consideration. First, all participants in the OA group had moderate to severe knee OA (KL grade 3 or 4) with pain ratings ≥3/10, and as such the findings cannot be generalised to knee OA populations with less severe OA. Second, although sample size used in this study is small, it is similar to many studies of cortical reorganization, in particular fMRI studies, and was sufficient to identify significant change in knee representation. Finally, as no x-ray or MRI imaging was undertaken for the ankles or elbows in either group, or the knees in the control group, we cannot exclude the possible presence of early pain-free OA changes to these joints in the current participant group.

## Conclusion

An anterior shift in the representation of the knee in the motor cortex and a switching of the arrangement of the knee and ankle representations was found in people with knee OA relative to disease-free controls. The results of this study indicate that poorer performance of a complex motor task is associated with more anterior placement of activation in the motor cortex both in people with and without knee OA. These results suggest there may be relationships between changes to motor control associated with knee OA and differing organisation of the motor cortex. Further investigation of whether there is a causal relationship between differing motor cortex organisation and the changes to motor control in knee OA is required. Better understanding of the causal relationships along with the current results indicating remodeling of the motor cortex associated with knee OA may provide direction for future treatments of knee OA as there are documented associations between treatments that contribute to normalisation of cortical organisation and improvement of symptoms in other chronic pain and musculoskeletal conditions.

## Abbreviations

BOLD: blood oxygenation level dependant
COPE: contrasts of parameter estimates
FLIRT: FMRI Image Laboratory Linear Registration Tool
fMRI: functional magnetic resonance imaging
FWHM: full width half maximum
GLM: general linear model
KL: Kellgren and Lawrence
MNI: Montreal Neurologic Institute
MRI: magnetic resonance imaging
MVC: maximum voluntary contraction
OA: osteoarthritis
RMS: root mean squared
WOMAC: Western Ontario and McMaster Universities Arthritis Index
